# Effectiveness of Surface Treatment on Bonding Performance of Starch-Based Aqueous Polymer Isocyanate Wood Adhesive

**DOI:** 10.3390/polym15040988

**Published:** 2023-02-16

**Authors:** Xinyue Ma, Junyou Shi

**Affiliations:** 1 Faculty of Bioscience Engineering, Jilin Agricultural Science and Technology University, Hanlin Rond, Jilin City 132101, China; 2 College of Materials Science and Engineering, Beihua University, Jilin City 132013, China

**Keywords:** surface treatment, starch-based API, adhesive properties, hygrothermal aging, wood adhesive

## Abstract

The surface of a bonding material plays a key role in the bonding performance of an adhesive. Herein, we evaluated the effect of substrate surface treatment methods (sandpaper polished, chemical oxidation, and coupling agent) on the adhesive properties of starch-based aqueous polymer isocyanate (API) wood adhesive during hygrothermal aging. The birch substrate was processed with three different surface treatments, and the change of surface was analyzed by X-ray Photoelectron Spectroscopy (XPS), Fourier Transform Infrared spectroscopy (FT-IR), and Energy Dispersive Spectroscopy (EDS) methods. The results showed that the surface treatment had a great influence on the change of the shear strength of glued wood under hygrothermal conditions, and the silane coupling agent treatment could effectively reduce the decrease in the compressive shear strength of the adhesive. An XPS analysis indicated that the chemical oxidation modified wood surface polarity, and the coupling agent treatment in the wood surface formed a transition layer. After hygrothermal aging treatment, due to the different surface treatment of adhesive joint surface binding energy, the internal water absorption rate of starch-based API adhesives exhibited different failure modes of the adhesive joint. These findings indicate that the surface treatment effectively improved the durability of the adhesive joints.

## 1. Introduction

Due to the constant changes in the application environment, there is a problem of moisture absorption and hydrolysis in the process of bonding, so the research on the resistance to hygrothermal aging is more complicated and varied. The surface of the wood is affected by changes in temperature and humidity, and therefore one of the effective ways to increase the durability of wood bonding joints is by increasing the interface strength of the wood surface [1,2]. Many of the surface treatments of wood have been used to modify the surface of wood, such as sandpaper grinding, chemical oxidation, coupling agent, microwave and plasma treatment [3]. Generally speaking, the surface treatment can improve the durability of the bonding joint. By chemically oxidizing the surface of the metal material, it can form the microcellular honeycomb structure on the metal surface, which greatly increases the bonding area and improves the bonding strength and durability. The non-metallic material surface can also be oxidized, which can produce more polar groups and subsequently improve bonding properties and durability [4,5]. The coupling treatment can form a transition layer between the adhesive material and the adhesive, which also helps to improve the durability of the bonded joint. Other treatments, such as plasma treatment, microwave treatment and irradiation treatment, are more suitable for non-metallic materials, so the surface polarity of the material is greatly improved, and therefore obtains a good bonding effect [6,7,8].

The durability of adhesives greatly affects the aging resistance of wood composites. However, research on the durability of adhesives containing isocyanate is limited. Generally, durability experiments are divided into accelerated experiments and exposure experiments, especially accelerated aging experiments, which are more important in revealing the aging resistance of a specific degradation factor. Umemura [9] studied the durability of isocyanate adhesives exposed to heating conditions through accelerated experiments. The results show that the activation energy increases with the increase of mass loss. The change of activation energy indicates that the degradation reaction of resin is not a single basic process. As for the durability of wood adhesives, the two important factors affecting their degradation are heat and humidity. In many cases, heating durability tests are performed at temperatures above 100 °C, whereas moisture tests are performed below 100 °C. Therefore, it is difficult to study the interaction of these degradation factors. In order to solve this problem, high pressure steam was used for research [10]. Bowditch [11] et al., studied the influence of surface treatment on the durability of adhesives and showed that water can gradually penetrate into all bonding interfaces along the surface of bonding materials, thus reducing the bonding strength. This effect is mainly due to the fact that the adsorption capacity of very polar water molecules and the surface of the bonded material is greater than that of the adhesive and the surface of the bonded material. Wylde [12] et al., calculated the moisture diffusion coefficient of metal joints bonded by epoxy resins under the condition of wet and thermal aging by using the element analysis method. Knox [13] et al., studied the change behavior of microscopic morphology and interfacial chemical bonds of bonded joints after long-term wet and thermal aging, and the results showed that the diffusion of water in adhesives is not only a physical effect but also a hydrolytic one.

The influence of the shear strength of the bonded joint with the aging condition and the effect of various surface treatment methods on the durability of the bonded joint has been studied extensively, such as the diffusion coefficient of the moisture in the adhesive joint, and the effects of different surface treatment methods on the durability of the bonded joint in terms of adhesive strength, surface microstructure and chemical bond change were investigated. Related research has been focused on the technical indicators of adhesives to measure changes with the aging conditions and the impact on the bonding strength [14,15,16], but for different surface treatment methods on the durability of adhesive bonding joints, bonding joint surface morphology with the aging of the law of change and other theoretical studies have been rare.

## 2. Materials and Methods

### 2.1. Materials

The birch specimens with moisture content from 8% to 10% and starch-based aqueous polymer isocyanate wood adhesive were provided by Jilin Chenlong Biomass Materials Co. Ltd., Jilin City, China, and the preparation of the bonding joint was according to the Japanese industrial standard (JIS K6806) [16]. The other chemicals of analytical grade were purchased from Sinopharm Chemical Reagent Beijing Co., Ltd. Beijing, China, and used as received without further purification.

### 2.2. Surface Treatment

In this experiment, the sandpaper of 1000 mesh was selected, and the shear strength was measured by pressing glued wood to optimize the sandpaper count. Sodium hypochlorite was used as the chemical oxidant at a concentration of 20%, and then the surface of the birch substrate was dried in a drying oven (40 °C). The glued wood was then prepared, and its compressive shear strength was measured. A silane coupling agent can improve the polarity of the wood surface, thereby increasing the bonding strength of its adhesive products. In this experiment, the surface of the wood was coated, baked in the drying oven (40 °C), and then pressed to measure the compressive strength. The concentration of KH550/ethanol solution was 3%.

### 2.3. Hygrothermal Treatment

A hygrothermal treatment was performed in the alternating humidity and heat test chamber (Nanjing Anai Experimental Equipment Co., LTD., Nanjing, China). The relative temperature and humidity were respectively set to 85 °C and 95% with different aging times.

### 2.4. Compressive Strength Test

Compressive strength specimens were prepared and tested according to the Japanese industrial standard (JIS K-6806) [16].

### 2.5. Water Absorption Rate Calculation

Because the EDS results are relative, the element analysis method is used to calculate the proportion of carbon and oxygen of starch-based API after curing. EDS is used to calculate the increment of oxygen in the process of hygrothermal aging, and then deduces the water absorption rate of the adhesives. The formula is as follows:

Water absorption (%) = (W × R − W_0_) × M_H2O_/M_O_ (1)

In the formula:

W—The proportion of the carbon and oxygen elements in the adhesives of adhesive adhesives;

R—The content of the oxygen element and the carbon element in the adhesive before the wet and heat aging;

W_0_—The content of the oxygen element in adhesive before wet and heat aging;

M_H2O_ and M_O_—The molecular weight of water and oxygen.

### 2.6. X-ray Photoelectron Spectroscopy

XPS is mainly used to detect the chemical composition and functional group changes of the birch substrate after the different methods of surface treatment. XPS test equipment and conditions: American thermoelectric ermo ESCALAB250 (AeKd hγ = 1486.6 eV), pass 20 eV, vacuum 3.2 × 10^−7^ Pa, etching energy 3 kV, focusing voltage 3 kV, target source Are ion.

### 2.7. FT-IR Analysis

All infrared spectra were obtained using an FT-IR(VERTEX70) spectrometer produced by the Bruker Company (Ettlingen, Germany) using the KBr pellet method, and were recorded with an average of 16 scans at a resolution of 4 cm^−1^ [16].

### 2.8. Energy Dispersive Spectroscopy Analysis

EDS was used to observe the change of oxygen atoms in the adhesive in the bonded joint after the hygrothermal aging treatment, and the water absorption was calculated. The change of oxygen content in the fracture of the bonded joint was detected, and the failure mode of the bonded joint was deduced.

## 3. Results

### 3.1. Effect of Substrate Surface Treatment Method on Bonding Strength

**Figure 1:**
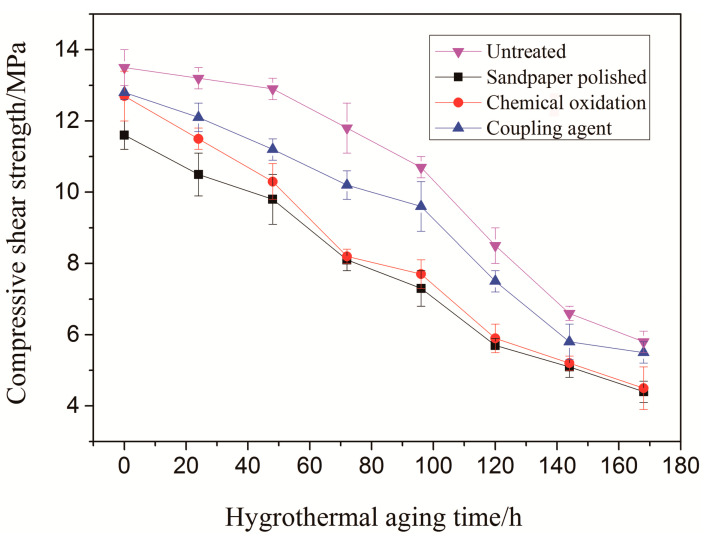
Compression shearing strength change of adhesive joints for the different surface treatments under hygrothermal aging.

The birch substrate was treated with three kinds of surface treatment methods, namely, sandpaper grinding, chemical oxidation and silane coupling agent treatment. The main agent and the crosslinking agent were prepared by adjusting the adhesive of 100:18 quality and pressing the birch wood. (50 °C, RH98%) for seven cycles; each cycle was 24 h, the shear strength of the glued wood was measured for each cycle, and the change of the shear strength of the bonded joint is shown in Figure 1. Error bars represent the standard deviation of the mean of at least three different experiments.

As can be seen from Figure 1, under different surface treatment methods, the shear strength variation trend of the adhesive joint is different. The surface of the substrate after treatment and the glued wood shear strength reduction rate is relatively slow. The tensile strength of the adhesive joint of the sandpaper has a linear trend in the initial period. Although the sandpaper grinding treatment improves the contact area, the adsorption energy and desorption energy of the moisture between the adhesive and the binder are not changed. Due to the weak adhesion of the adhesive and wood, moisture is easily diffused from the adhesive interface into the interior of the adhesive, so the shear strength decreases rapidly. When the hygrothermal aging time reaches 144 h, the shear strength is mainly the mechanical bond between the adhesive and the wood, so the shear strength decreases slowly. The trend of compressive shear strength of the sandpaper grinding treatment is similar to that of the untreated sample. The trend of chemical oxidation and sandpaper grinding treatment is basically the same, but the degree has slowed. This is due to a small number of polar groups formed on the surface of the adhesive materials treated with chemical oxidation and formed microcracks, which improved the adhesion of the adhesive and the substrate. In contrast, the shear stress intensity curve of the silane coupling agent treatment is different from that of the former two. The compressive shear strength is kept constant when the wet compressive aging is carried out for 24 h. During this period, due to the presence of the coupling agent, the diffusion of moisture in the bonding interface is suppressed, and these small amounts of water penetrate into the adhesive, and only the plasticizing effect is made to keep the compressive shear strength constant. When the hygrothermal aging time is extended to 48 h, the water gradually spread to the inside of the adhesive, and the shear strength of the bonding joints began to decline. After 144 h of hot and humid aging treatment, the bonding strength of the bonded joint becomes very low, and the bonding strength is mainly the mechanical lock structure between the adhesive and the wood, so the shear strength decreases slowly. It has been shown that the surface treatment has a great influence on the change of the shear strength of glued wood under the condition of hot and humid aging, and the silane coupling agent treatment can effectively reduce the decrease in the compressive shear strength of the API adhesive.

### 3.2. XPS Analysis of Substrate

**Figure 2:**
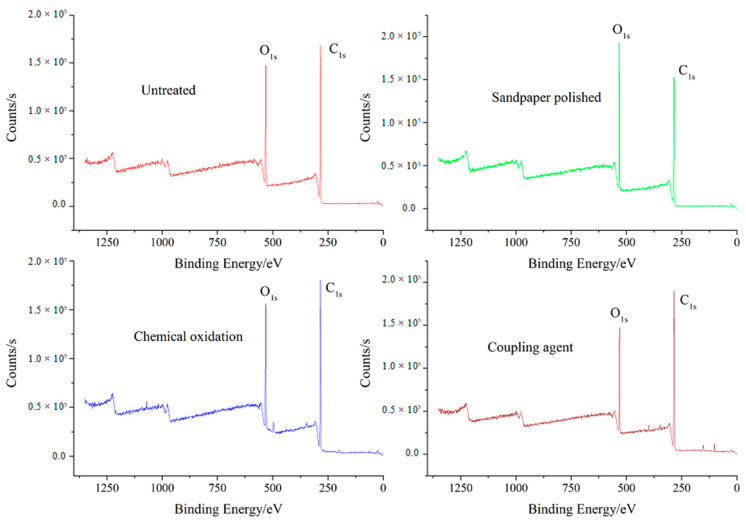
Survey XPS spectra of wood surface for the different surface treatment methods.

The composition of the wood is mainly C, H, and O. In this experiment, the birch substrate was subjected to different surface treatments. Because the depth of treatment was very shallow, the wide scan of the XPS can mark the inner electron bonding energy of all the elements in the wood (except H and He), so that the specific binding energy of each element can be used to identify the wood surface element composition and relative content. The XPS wide scan pattern of the surface of the different surface treatment methods is shown in Figure 2. The composition of the wood surface elements is shown in Table 1.

From Table 1, the content of C element in the wood after the surface treatment was decreased, the O element content was increased, and the ratio of the atomic concentration of O/C was increased. In addition, the sandpaper treated birch substrate only slightly increased the bonding area, and the element content saw a slight change. The oxygen content of the substrate surface treated by chemical oxidation increased from 22.03% to 23.35%, indicating the surface oxidation reaction of the wood. Meanwhile, the oxygen content of the birch substrate treated by the silane coupling agent was increased from 22.03% to 25.51%, which indicated that the silane coupling agent and the polar groups on the wood surface underwent an oxidation reaction followed by the introduction of oxygen-containing groups.

**Figure 3:**
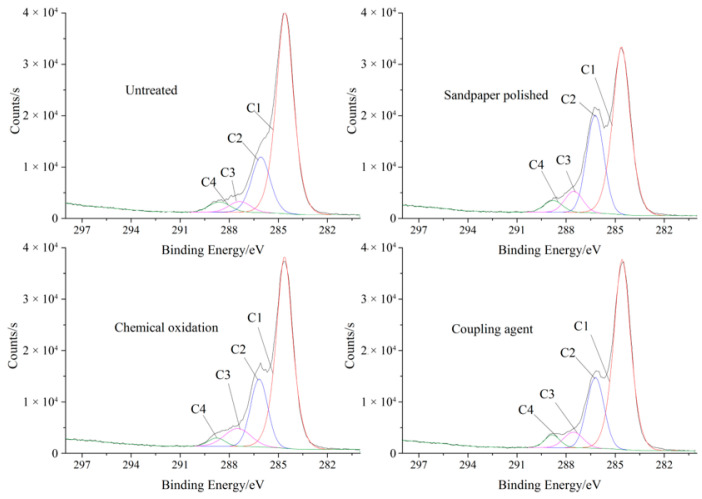
XPS spectrum of wood C1s for the different surface treatment methods.

The change of chemical structure in the surface treatment can be analyzed by using the change of carbon atom binding energy and intensity to determine the existence and relative content of carbon atoms in the wood. In order to characterize the change of the main groups on the surface of the birch substrate, the distribution of the carbon content in different chemical environments was analyzed. The C1s on the birch substrate were subjected to peak treatment by spectral curve fitting. The spectra of the C1s peak of the surface of birch treated with different treatment methods are shown in Figure 3, and the position and peak area data of the carbon atoms are shown in Table 2. Due to the influence of the electronic effect, the measured binding energy is lower than the standard value, and the calibration is basically within the standard value range.

From the experimental results, the birch substrate contains four different carbon atoms, namely C1, C2, C3 and C4, although primarily C1 and C2. The combination of carbon has undergone significant changes after the treatment. The content of C1 and C4 in the surface of the substrate is decreased, and the content of C2 and C3 is increased, which indicates that the structure of carbon-containing functional groups on the wood surface changed. It is possible that after the surface treatment, the carbon atoms contained in the wood surface are connected to the polar hydroxyl or ester groups, where the high oxidation morphology increased the electron binding energy.

**Figure 4:**
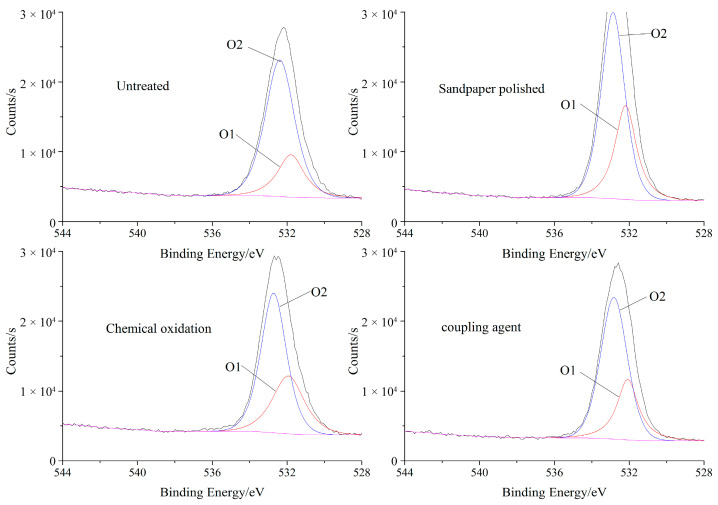
XPS spectrum of wood O1s for the different surface treatment methods.

The oxygen atoms and carbon atoms in the wood cellulose are mainly single bonds, which are divided into O1 and have higher binding energy. However, the binding energy of oxygen in the form of double bonds with carbon is very low, and it is classified as O2. After the chemical oxidation and silane coupling agent treatment, the content of carbon and oxygen double bonds on the wood surface increased, so we studied the change of the O1s peak in XPS spectra. Figure 4 shows the position and peak area of the oxygen atoms in the O1s of the birch wood surface treated with different surface treatment methods, and the XPS data is shown in Table 3.

It can be seen from the experimental results that the peak area of O1 increased and the peak area of O2 decreased, indicating that the hydroxyl groups in the cellulose and hemicellulose were oxidized after the surface treatment, and the silane coupling agent treatment causes the polar groups on the wood surface to undergo a chemical reaction, resulting in an increase in the oxygen-containing functional groups.

### 3.3. Birch Surface Treatment FT-IR Analysis

**Figure 5:**
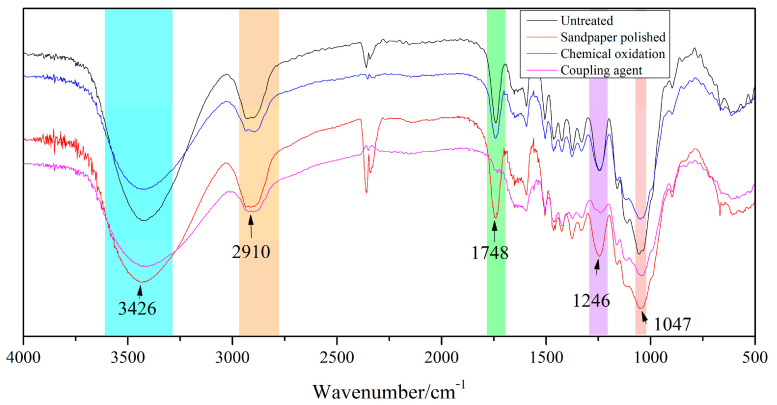
FT-IR spectrum of wood surface treated by the different treatment methods.

The change of the main groups and the improvement of the interface properties after the surface treatment of the birch substrate are of great significance to improve the aging resistance of the glued materials. The FT-IR spectra of untreated and surface-treated birch substrates are shown in Figure 5. It can be seen from Figure 5 that the spectra of the four samples have characteristic absorption peaks at 1047.04 cm^−1^, 2910.30 cm^−1^ and 3426.30 cm^−1^, respectively. After chemical oxidation and coupling agent treatment, the wood surface absorption peak disappeared at 2364.51 cm^−1^, while the sandpaper polished surface was enhanced here. In addition, the absorption peaks of the surface of the wood treated by the coupling agent at 1748.38 cm^−1^ and 1246.65 cm^−1^ also disappeared, indicating that the introduction of certain groups and the groups of the wood itself occurred after chemical oxidation and coupling agent treatment.

### 3.4. Effect of Surface Treatment on the Water Absorbency of Adhesive in Bonding Joint

**Figure 6:**
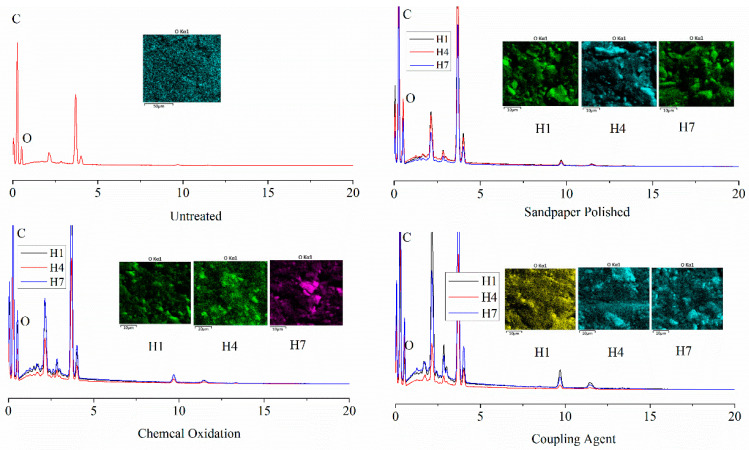
Oxygen element spectra of adhesive on the joint in different surface treatment methods.

A bonded joint was prepared using a surface treated birch substrate and the starch-based API adhesive. The adhesive joint was then treated in a hygrothermal aging environment (temperature: 50 °C, relative humidity: 98%) for a total of seven cycles. EDS was used to observe the change of oxygen distribution and content on the surface of the adhesive joint. The EDS spectrum of the bonding joint with different surface treatment methods is shown in Figure 6, and the oxygen content is shown in Table 4.

From the experimental results, the surface oxygen distribution and the oxygen content of the bonded joint changed after the hygrothermal aging treatment. The content of oxygen element in the API adhesive of the bonding joint was increased, which varied among different surface treatment methods. With the hygrothermal aging cycle extended, the oxygen content which is treated with the same method is also gradually increased. There is an isochronous relationship between the surface treatment method and the hygrothermal aging cycle.

**Figure 7:**
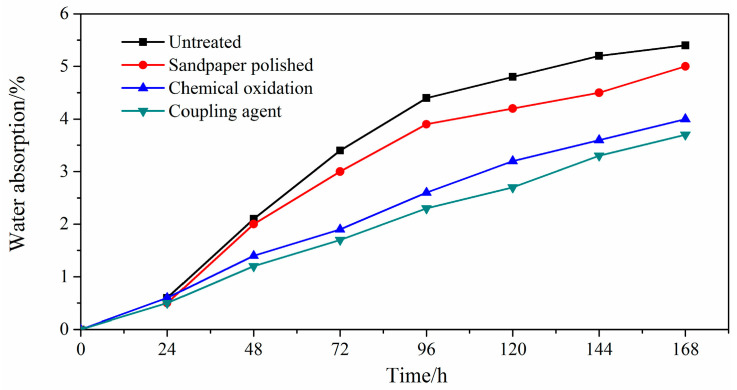
Relation between the water absorption of the bonding joint and the hygrothermal aging cycle.

Surface treatment can improve the surface bonding energy of glued products, which can be slowed by the penetration rate of water. The oxygen content of the adhesive in the adhesive was measured by EDS at 50 °C and RH98% under the condition of hygrothermal aging. The water absorption of the adhesive in the glued wood treated by different surface treatment methods was calculated according to the formula. The relationship between heat aging time is shown in Figure 7.

As can be seen from Figure 7, after the hygrothermal aging, the starch-based API adhesive joint water absorption in the first cycle increased very slowly. When the aging treatment reached 48 h, the adhesive joints increased rapidly owing to the water that had penetrated into the bonding joint. The water absorption of the adhesive in the bonding joint of the untreated and polished sandpaper is higher than the other two treatment methods, mainly because the lower bonding interface energy made the water permeate faster. After 120 h, the water absorption rate of the four methods slowed down, and the trends were similar. By this time, the water has been through the bonding interface into the internal of the adhesive. As the adhesive structure is the same, the water permeate velocity from the outer surface of the adhesive to the internal is the same, but from the bonding interface to penetrate in the internal is different. The overall degree of moisture spread into the interior of the adhesive is still different, and this leads to the low bonding energy of the untreated and sandpaper polished adhesive joints, showing the faster trend of the automatic fracture, combined with the higher bonding energy of the chemical oxidation and coupling agent treatment of the adhesive joints, which will delay their breaking.

**Figure 8:**
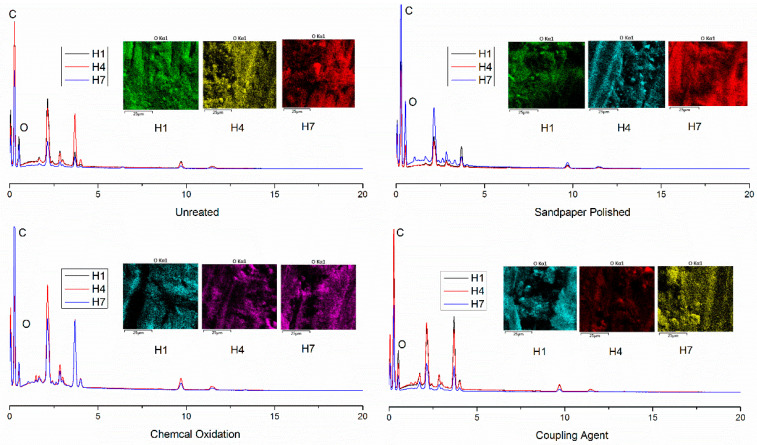
Distribution of oxygen on the broken surface of the bonding joint under different hygrothermal aging.

It is possible to determine the damaged form of the adhesive joint prepared by the starch-based API adhesive by analyzing the content of the surface element after the failure of the bonded joint by EDS. The oxygen content of the bonded joint is shown in Table 5 and the distribution form is shown in Figure 8.

It can be seen that the oxygen content of the surface of the adhesive is 28.3% at 50 °C and RH 98%, which is determined by its chemical structure. When the adhesive joint was hygrothermally treated for 48 h, the oxygen content of the treated and sandpaper polished adhesive joint was 27.1% and 26.9%, respectively, which was lower than the oxygen content on the surface of the adhesive. The cohesive strength of the adhesive is still higher than the birch’s cohesive strength and interfacial bonding strength. Therefore, the damage of the bonding joint occurs inside the birch, so the oxygen content is less than that before the hot and humid aging. While the content of oxygen in the fracture of the adhesive joint treated by chemical oxidation and the coupling agent is increases, it is still lower than the oxygen content of the adhesive surface at room temperature. It has been shown that the cohesive strength of the adhesive is the same as the interfacial adhesion strength of the birch substrate with API adhesive, and the failure mode is mixed destruction. With the hygrothermal aging, the fracture oxygen content of untreated and polished sandpaper treatment of the bonding joint is increasing, and it is higher than the oxygen content of the adhesive. During this period, the cohesive strength of the adhesive is lower than that of the birch material and the interfacial bonding strength, so the failure of the bonding joint occurs inside the adhesive. Because of the water absorption of the bonding joint, the oxygen content increased, and it is higher than the surface oxygen content of the adhesive before the aging. After 72 h of the chemical oxidative treatment and 96 h of the coupling agent treatment, the oxygen content of the joint fracture was higher than that of the adhesive at room temperature, and then the failure mode of the adhesive joint was changed. There is also an isochronal relationship between the oxygen content of the fracture surface of the bonded joint and the different surface treatment methods.

## 4. Conclusions

The analysis methods of XPS, FT-IR and EDS showed that after chemical oxidation and coupling agent treatment, the content and valence state of the carbon element and the oxygen element of the birch substrate were significantly changed. After the hygrothermal aging treatment, due to the different surface treatment of the adhesive joint surface binding energy, the internal water absorption rate of starch-based API adhesives shows the different failure mode of the adhesive joint. The use of the surface treatment effectively improved the durability of the adhesive joints.

## Figures and Tables

**Table 1:** Chemical element composition of wood surface for the different surface treatment methods.

Surface Treatment Method	Element Content/%		(O/C)/%
	C	O	
Untreated	77.97	22.03	28.25
Sandpaper polished	77.45	22.25	28.73
Chemical oxidation	76.07	23.35	30.70
Coupling agent	74.49	25.51	34.25

**Table 2:** Binding energy and C1 peak area of birch surface by the different surface treatment methods.

Sample	Untreated		Sandpaper Polished		Chemical Oxidation		Coupling Agent	
	Binding Energy/eV	Content/%	Binding Energy/eV	Content/%	Binding Energy/eV	Content/%	Binding Energy/eV	Content/%
C1	284.60	53.88	284.61	42.55	284.62	50.41	284.57	51.16
C2	286.07	15.62	286.22	23.25	286.17	17.50	286.21	18.65
C3	287.36	3.33	287.53	5.19	287.49	7.10	287.55	4.57
C4	288.60	3.21	288.79	2.90	288.77	2.10	288.84	3.02

**Table 3:** High resolution O1s XPS data of wood surfaces for the different surface treatment methods.

Sample	Untreated		Sandpaper Polished		Chemical Oxidation		Coupling Agent	
	Binding Energy/eV	Content/%	Binding Energy/eV	Content/%	Binding Energy/eV	Content/%	Binding Energy/eV	Content/%
O1	532.39	16.71	532.87	16.21	532.72	14.96	532.82	15.53
O2	531.79	5.01	532.19	8.54	531.93	7.93	532.08	6.34

**Table 4:** Oxygen content of bonding joint at different surface treatment methods.

Aging Time/h	Oxygen Content/%			
	Untreated	Sandpaper Polished	Chemical Oxidation	Coupling Agent
0	28.3	28.2	29.1	29.6
24	28.8	28.6	29.6	30
48	30.2	30	30.4	30.7
72	31.3	30.9	30.8	31.1
96	32.2	31.7	31.2	31.6
120	32.6	32	31.8	32
144	32.9	32.3	32.0	32.4
168	33.3	32.7	32.4	32.9

**Table 5:** Oxygen content of fracture surface for the bonding joint under different hygrothermal aging.

Hot and Humid Aging Time/h	Oxygen Content/%			
	Untreated	Sandpaper Polished	Chemical Oxidation	Coupling Agent
0	28.3	28.3	28.3	28.3
24	24.7	24.1	23.6	22.8
48	27.1	26.9	24.4	23.5
72	30.3	29.1	26.7	24.7
96	31.1	29.9	29.5	25.6
120	32.5	31.5	30.2	29.9
144	34.6	32.8	31.1	30.4
168	36.1	34.2	32.3	31.2

## Data Availability

The data presented in this study are available on request from the corresponding author.
